# Platelet Membranes Coated Gold Nanocages for Tumor Targeted Drug Delivery and Amplificated Low-Dose Radiotherapy

**DOI:** 10.3389/fonc.2021.793006

**Published:** 2021-11-24

**Authors:** Mingzhu Chen, Ping Wang, Dazhen Jiang, Zhirong Bao, Hong Quan

**Affiliations:** ^1^ Key Laboratory of Artificial Micro- and Nano-Structures of Ministry of Education, School of Physics and Technology, Wuhan University, Wuhan, China; ^2^ Department of Molecular Pathology, Henan Cancer Hospital, Affiliated Tumor Hospital of Zhengzhou University, Zhengzhou, China; ^3^ Department of Radiation and Medical Oncology, Hubei Key Laboratory of Tumor Biological Behaviors, Hubei Cancer Clinical Study Center, Zhongnan Hospital of Wuhan University, Wuhan, China

**Keywords:** platelet membranes, gold nanocages, drug delivery, low-dose radiotherapy, cisplatin

## Abstract

Continuous high doses of radiation can cause irreversible side effects and radiation resistance; thus, advanced radiosensitizers are urgently needed. To overcome this problem, we developed a nano platelet radiosensitization system (PCA) by coating the chemotherapeutic drug cisplatin (CDDP) loaded gold nanocages (AuNs) within the platelet membrane. The developed PCA system may enable AuNs to have immune escape and targeting capabilities. After administration, PCA will actively target tumor cells and avoid being cleared by the immune system. Subsequently, CDDP, which destroys tumor cell DNA, can not only kill tumor cells directly but also combine with AuNs, which deposit radiation energy into tumor tissues, reducing RT resistance. *In vivo* and *in vitro* studies revealed that the combination of PCA with RT (2Gy) efficiently inhibits tumor proliferation without causing side effects such as inflammation. To conclude, this is the first attempt to use platelet membranes to correctly transport AuNs while also accomplishing low-dose RT, which could help AuNs-based tumor RT become more effective.

## Introduction

Despite current scientific advancements, cancer, which can strike at any age and affect anybody, remains a severe threat to human life and health (1–3). Radiotherapy (RT) is widely used to treat cancer patients, either alone or in combination with other cutting-edge treatments (4). RT is based on the use of high-energy X-rays or gamma rays to cause radiation-induced DNA damage and triggers the generation of reactive oxygen species (ROS) in increased concentrations (5). Both radiation-induced DNA damage and ROS generation that exceeds the cell’s ability to neutralize these free radicals result in apoptosis, which reduces tumor size (6). However, while RT kills tumor cells, it also kills adjacent cells and tissues in the human body (7). RT produces ROS in a dose-dependent manner, with higher doses resulting in better therapeutic outcomes (8). However, high-dose RT might cause systemic effects such as fatigue, loss of appetite, bone marrow suppression, RT-induced secondary and primary malignancies, and infertility, as well as local RT damage (9–11). Local liver damage can lead to changes in activity or, in the worst-case scenario, liver failure (12). Therefore, there is a definite need to find novel methods to improve RT effectiveness in cancer patients.

The emergence of nanoplatforms has enabled more and more novel nanomaterials to be delivered to tumor tissues to accomplish radiosensitization while reducing systemic toxicity, as a result of the integration of nanotechnology in the area of cancer treatment (13). MoS_2_-based nanomaterials and copper selenide nanomaterials have been explored to realize radiosensitization (14, 15). These studies provide new insights for the selection of radiotherapy sensitizers. Gold nanomaterials, such as gold nanorods, gold nanostars, gold nanocages, and other High-Z nanomaterials, are the most common of these (16–18). Gold, a High-Z nanomaterial, can enhance the deposition of radiation energy in the tumor and improve the effectiveness of RT (19). For example, Liu et al. developed a new type of gold nanostar probe that can simultaneously realize tumor imaging and photothermal therapy *in vivo (* 20). Unlike other gold nanomaterials, gold nanocages have the excellent drug-carrying ability. Zhu et al. (21) designed paclitaxel-loaded gold nanocages and wrapped them with red blood cell membranes to achieve combined anti-tumor effects *in vitro*. However, no article has yet been published that describes the usage of gold nanocages to produce radiosensitization.

For a long time, people have known that platelets play an important role in hemostasis through clotting and blood vessel damage (22–24). Platelets can also interact with tumor cells by directly binding or secreting cytokines. Taking advantage of the natural pathophysiological affinity and targeting ability of platelets to tumors, a biomimetic drug delivery system based on platelet membranes has also been developed for tumor treatment (25, 26). Compared with artificial drug delivery platforms, they are less likely to cause clearance reactions, so vesicles administered with the platelet membrane itself provide a better prospect for drug delivery (27, 28). Platelet membranes are easier to penetrate from blood vessels to tumor tissues for precision medication administration or nanomaterial delivery due to the properties of membrane proteins. The oxidative stress amplifying drug cinnamaldehyde (CA) coated platelet membrane hybrid nanosystem (PSCI) was developed by Huang et al. using the peculiarities of platelet membranes (PM) to transport CA to tumor tissues and achieve up to 90% tumor growth suppression (22). Therefore, it stimulates us to add gold nanocages to PM to overcome radiation resistance and immune clearance.

**Scheme 1:**
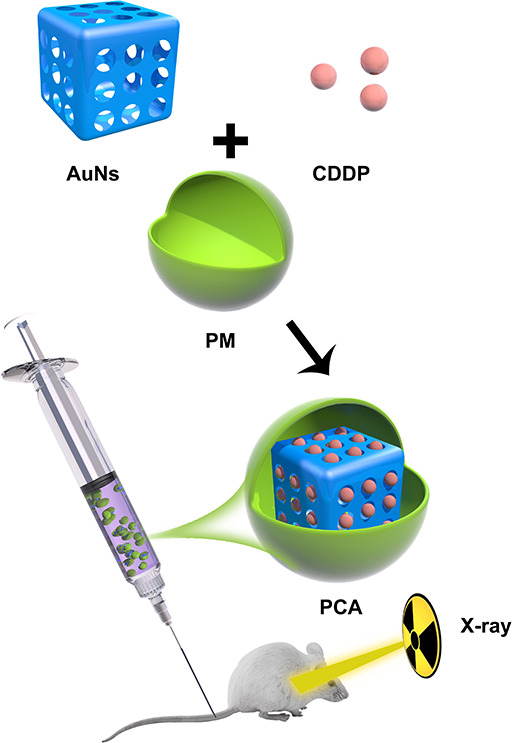
Schematic illustration of platelet membranes coated gold nanocages for tumor targeted drug delivery and amplificated low-dose radiotherapy.

**Figure 1:**
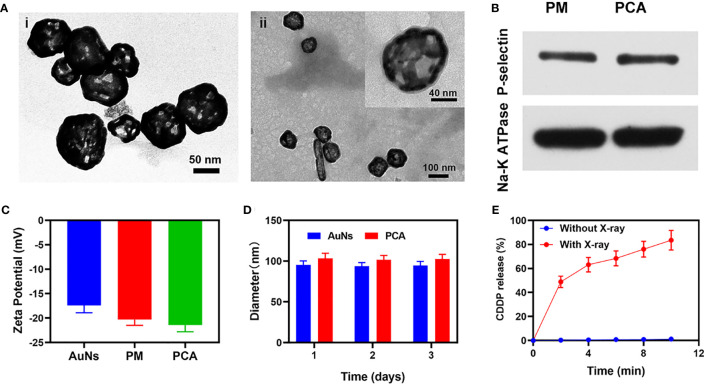
**(A)** TEM image of (i) AuNs and (ii) PCA. Insert: single PCA with high magnification. **(B)** The expressions of the key protein P-selectin on PCA with PM as a control. **(C)** Zeta potential of AuNs, PM, and PCA. **(D)** Statistical graph of the measured diameter of AuNs and PCA. **(E)** CDDP release profiles under different conditions.

For the first time, a study on the combined application of AuNs and PM to improve low-dose RT was published in this study. A composite nanosystem PCA was constructed by coating AuNs containing the anticancer drug cisplatin (CDDP) with PM (**Scheme 1**). The platelet membrane can protect CDDP and AuNs, prevent these medicines from leaking in advance, and successfully improve the blood circulation time of AuNs in the body because PCA has targeting and radiosensitization capabilities. At the same time, it helps the drug overcome the renal effect and immune clearance, and actively targets tumor cells. Subsequently, CDDP, which destroys tumor cell DNA, can not only directly kill tumor cells, but also can combine with AuNs that deposit RT energy to tumor tissues to achieve high-efficiency sensitization RT. It needs to be pointed out that in the treatment window, we only need 2 Gy of RT and PCA synergistic treatment to obtain a strong radiation sensitization effect. PCA system provides a higher biological potential for long-term use. Finally, these findings broaden the use of gold nanocages and provide new insights for the development of platelet membrane-based tumor treatment systems.As shown in **Scheme 1**, we firstly extracted platelet vesicles (PV) from platelets. The AuNs and CDDP were co-coated with PV to obtain a PV-coated composite nano-system (PCA). **Figure 1A** shows a transmission electron microscopy (TEM) image of the prepared AuNs and PCA. PCA exhibited a gray-scale platelet membrane (PM) of about 8.5 nm. PCA and platelet membrane showed the same P-selectin protein profile, as shown in **Figure 1B**, indicating that platelet membrane proteins are completely retained in PCA. These results showed that we have successfully prepared AuNs and composite material PCA. Platelet membranes have a lot of potential in the field of substance delivery because of this characteristic, which makes them far superior to standard drug delivery methods like liposomes or red blood cell membranes that don’t target anything (29, 30). P-selectin in platelet membranes can specifically recognize corresponding ligands on the surface of tumor cells, and these interactions give PCA the ability to actively target tumor cells. None of these materials increase the penetration of the substance into the tumor tissue. The successful coating of PV allows the poorly targeted CDDP and AuNs to stay in the bloodstream for longer, evade immune system attacks, and actively target tumor cells. And we continued to measure the zeta potential of different materials, as shown in **Figure 1C**. During the three days, the particle sizes of AuNs were 95.4 ± 4.8 nm, 93.8 ± 4.2 nm, and 94.5 ± 5.1 nm, respectively, while the particle sizes of PCA were 103.4 ± 6.2 nm, 101.7 ± 5.2 nm, and 102.5 ± 5.7 nm (**Figure 1D**), which also indicates that both AuNs and hybrid PCA have good stability and can be used in subsequent biological experiments. The drug releasing ability of PCA have been investigated, as shown in **Figure 1E**, PCA hardly releases CDDP without X-ray exposure (**Figure 1E**). Once irradiated by X-rays, the platelet membrane will be damaged, and gradually release the drugs. The drug loading efficiency (DLE) was determined using high-performance liquid chromatography (HPLC). DLE was 94.6 ± 5.2%. These results suggest that PCA has a PM structure and that would be released from 4T1 tumor cells *via* extracellular action to change the tumor micro-environment.

**Figure 2:**
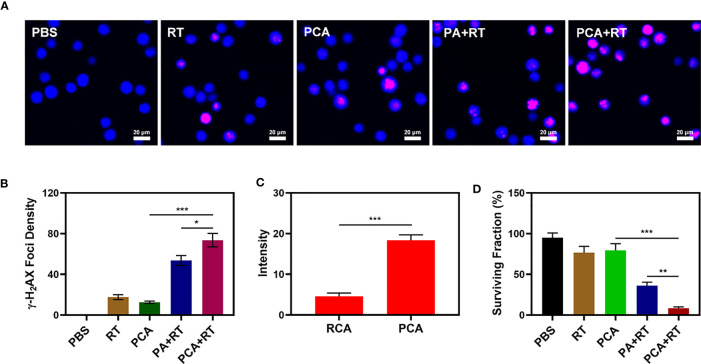
The CLSM images of 4T1 cells with different treatments. The nuclei were stained with DAPI (blue) and DSBs were stained with γ-H_2_AX (red). **(B)** Average fluorescence intensity of γ-H_2_AX from figure **(A)**. **(C)** Co-localization of Lyso-Tracker Green FM (blue) and Dil (red) for RCA and PCA over time in 4T1 tumor cells. **(D)** Colony formation assays were conducted using 4T1 cells treated with 2 Gy of radiation (n = 3). Significant differences among groups as calculated using the Student’s t-test. *P < 0.05, **P < 0.01, ***P < 0.005.

The PCA system is well-structured and characterized in terms of performance. We are actively conducting *in vitro* anti-tumor trials. Although AuNs and CDDP can change the ecological balance of the tumor microenvironment and therefore enhance the efficacy of radiation, they can only do so when they are located at the tumor tissue. The immune system can recognize the stimulation of various foreign invaders, and some of the stimulation can trigger the immune response, resulting in immunity, and inhibit the immune system from responding to other stimuli, resulting in tolerance (31, 32). However, the platelets are not attacked by the immune system, which is related to PM proteins. Unlike other drug delivery systems that do not have targeting capabilities rearranged nanomaterial-coated PCA may be directed to tumor cells, identified by cancer cells, and engorged to release nanomaterial. This internalization effect was tracked by staining RCA (red blood cell membrane comprising AuNs which have been loaded with CDDP) and PCA nanovesicles with Dil dye and co-incubation with 4T1 cells and staining with commercial lysosome Lyso-Tracker Green probe to verify the ability of PCA to be internalized by tumor cells *in vitro* (co-localization assay). After 2 h of incubation, it was clear that a significant quantity of PCA had been endocytosed, whereas RCA only had a partial measured fluorescence impact of Dil (**Figure 2C**), demonstrating that PM may be used as an excellent carrier of AuNs to target tumor tissues. When tumor cells are exposed to radiation, double-stranded DNA breaks (DSB) occur, providing insight into radiation sensitization. Measuring the fluorescence intensity of γ-H2AX is a good and intelligent technique to verify the formation of DSB after cell damage (33). Therefore, we detected H_2_AX foci in the nucleus after treatment in different groups. There was moderate DNA damage after low dose (2 Gy) RT. Notably, it is worth noting that 2Gy RT combined with PA only achieved about 53.6% γ-H2AX formation, while 2Gy combined with PCA achieved the best γ-H_2_AX foci formation, as high as 70.2% (**Figures 2A, B**). Compared with each experimental group, there was a uniform and significant difference, which was attributed to the targeting ability of platelet membrane and RT sensitization effect of CDDP and AuNs. Furthermore, the Colony formation assays test showed that the cell viability of the control group was almost unaffected, while RT combined with the PA group had moderate tumor growth inhibition (**Figure 2D**). Although Au can deposit RT rays to enhance its therapeutic efficacy, the single sensitization effect is limited. There were significant differences in tumor growth inhibition rates (the tumor inhibition rate reached 92.5%) between the PCA + RT system and the other experiment groups, indicating that CDDP in PCA mediated destroying DNA of tumor cells can effectively exert influence on cellular viability balance and thus enhance the effect of RT to achieve tumor growth inhibition. Together, these results drive continued exploration of anti-tumor efficacy *in vivo.*

**Figure 3:**
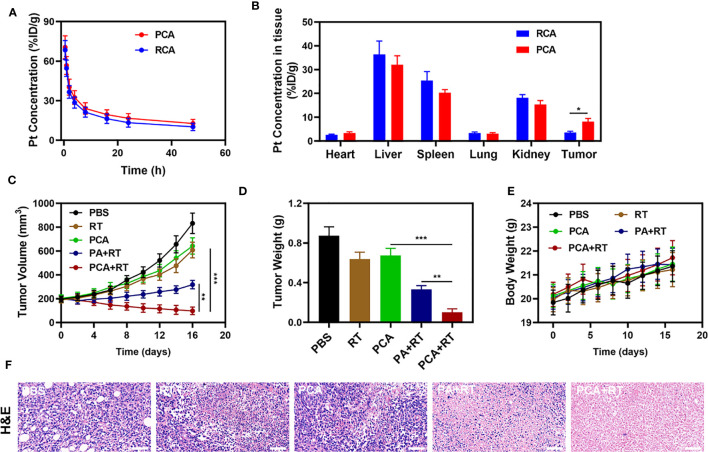
**(A)** Pharmacokinetic behavior of RCA and PCA in mice following i.v. administration. Data are presented as mean ± SD (n = 3). **(B)** Quantitative analysis of Pt biodistribution in tissues and tumors of tumor-bearing mice injected with RCA or PCA at CDDP dose of 5 mg/kg, respectively (n = 3). **(C)** Change in tumor-volume curves of 4T1 tumor-bearing mice after treatments (n = 5). **(D)** Changes in tumor weight following treatment (n = 5). **(E)** Body weight of 4T1 tumor-bearing mice as recorded every 2 days following treatment (n = 5). **(F)** Representative hematoxylin-eosin staining (H&E) stained tumor slice images of mice following treatment (n = 5), scale bars = 100 μm. Significant differences among groups as calculated using the student’s t-test. *P < 0.05, **P < 0.01, ***P < 0.005.

**Figure 4:**
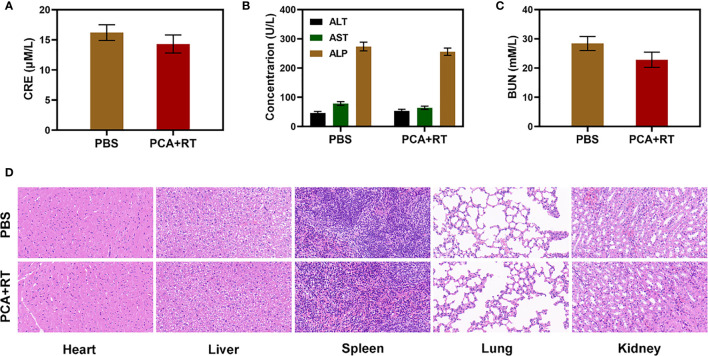
Result of *in vivo* safety experiments. Blood biochemistry data including kidney/liver function markers: **(A)** CRE, **(B)** ALT, ALP, and AST, and **(C)** BUN after various treatments. **(D)** Histopathological analysis results (H&E) stained images of the major organs, heart, lung, liver, kidneys, and spleen, of mice that were exposed to different treatments 16 days’ post-injection under laser irradiation.

Because an *in vitro* investigation of the tumor-killing effect revealed significant potential. We further explore *in vivo* intensification of PCA effects. Therefore, we conducted *in vivo* pharmacokinetic experiments to investigate the effect of platelet membranes on blood retention. Mice were intravenously injected with RCA or PCA at a dose of 1 mg CDDP/kg (**Figure 3A**). Next, we studied the biological distribution (**Figure 3B**). After 12 h of administration, the CDDP mainly accumulated in the liver and spleen of the RCA group of mice, while the PCA group showed good tumor targeting and low organ accumulation, further proving the targeting ability of the platelet membrane. Both PCA and RCA showed a stronger blood retention effect. Although the coating of erythrocyte membrane made AuNs and CDDP impervious to immune system attack, it lacked tumor targeting, and CDDP accumulation in tumor tissues was only marginally increased, with the PCA group having the most visible tumor accumulation. We next evaluated PCA-mediated anti-tumor efficiency in mice bearing 4T1 tumors. To investigate the primary effect of the PCA, BALB/c mice were subcutaneous injected with 1 × 10^6^ 4T1 cells into the right flank. The mice were grouped and treated when the primary tumor volume reached 200 mm^3^. Tumor-bearing mice were divided randomly into 5 groups (each group included 5 mice): 1) Control (PBS); 2) Radiotherapy (RT, 2Gy); 3) PCA; 4) PA + RT; 5) PCA + RT. The CDDP concentration was 1 mg/kg in groups 3 and 5. The AuNs concentration was 5 mg/kg in groups 3, 4, and 5. The treatment was conducted every 5 days for 15 days. During the 2 weeks treatment, the tumor volumes of the control group and RT treated group rose rapidly, as shown in **Figure 3C**. The PCA group also hardly inhibited tumor growth. The group of PA + RT also showed an almost moderate tumor suppressant effect. Although Au element can deposit RT rays to expand the RT therapeutic effect, the single sensitization effect is limited. When these nanomaterials are injected into the caudal vein, the PCA circulates to the tumor tissue and is endocytosed by tumor cells. As a result of the tumor cells’ endocytosis, AuNs are released into the tumor microenvironment (TME), having a therapeutic effect. Specifically, the PCA + RT system, which carried both CDDP and AuNs, achieved the most powerful therapeutic effect, with tumor volume growth curves almost completely suppressed during treatment. The tumor mass of mice was also consistent with the corresponding volume curve (**Figure 3D**). The treatment group did not gain or lose weight during the study, indicating that the treatment did not cause any significant systemic toxicity (**Figure 3E**). This is significant because many treatments are associated with extremely systemic toxicity, which is extremely detrimental to the future clinical application of the material. We took tumor tissue sections for H&E staining (**Figure 3F**) also confirmed that there was a large amount of cell necrosis in the PCA combined RT treatment group. As shown in **Figure 4**, during PCA activation treatment of mice, their vital organs (heart, liver, spleen, lungs, and kidney) were found to be healthy and free of inflammation and damage throughout the body. Furthermore, the liver and kidney indices were found to be normal as well. as many nanomaterials possess great therapeutic efficacy, they are also associated with systemic toxicity, which limits their future clinical applications (34, 35). The *in vivo* results demonstrate that this novel combined treatment not only achieves a good biological safety therapy but gives damage to tumor cell DNA, reinforces the effect of RT with profound PCA-enhanced therapy.

## Conclusion

In a nutshell, we developed a platelet membrane-based RT sensitizer to achieve enhanced RT results. The nano-agent AuNs and chemotherapeutic drugs encapsulated in PCA can deposit RT rays or damage tumor DNA to reduce RT resistance. Platelet membranes endow PCA with excellent targeting ability. These elements have a greater ability to induce tumor cell apoptosis. Both *in vitro* and *in vivo* results indicate that PCA system shows good tumor suppression. What matters is that the method we developed has no visible adverse effects during therapy. We will continue to investigate the biological applications of PM in combination with other new nanomaterials in the future, as well as combine additional nanotechnology to improve treatment approach.

## Materials and Methods

### Materials and Reagents

Deionized (DI) water was obtained by an 18 MΩ cm (SHRO-plus DI) system. Phosphate buffer solution (PBS) and bovine serum albumin (BSA) were purchased from Thermo-Fisher (USA). AuNs were obtained from XFNano (Nanjing, China)., 1’-dioctadecyl-3, 3, 3’, 3’-tetramethylindocarbocyanine perchlorates (Dil) was obtained from Sigma-Aldrich (USA). The γ-H2AX antibody was purchased from Abcam Company. The other reagents used in this work were purchased from Sinopharm Chemical Reagent (China) and Aladdin-Reagent (China).

### Preparation and Characterization of PLT-Vesicles (PM)

Platelets (PLTs) from whole blood were isolated through gradient centrifugation. 10 mL mice whole blood was centrifuged at 100 × g for 20 min with no brake. Afterward, the supernatant was centrifuged at 800 × g for 20 min. The PLTs were washed by PBS and centrifuged repeatedly. The preparation processes were monitored using a conventional microscope (IX71, Olympus, Japan). PLT membranes were derived by a repeated freeze–thaw process. Aliquots of PLT suspensions were first frozen at −80°C, thawed at room temperature, and pelleted by centrifugation at 4000 × g for 3 min. After three repeated washes with PBS mixed with protease inhibitor tablets, the pelleted PLT membranes were suspended in water, sonicated in a capped glass vial for 5 min using a bath sonicator at a frequency of 53 kHz and a power of 100 W, and then extruded sequentially through 400 and 200 nm polycarbonate porous membranes on a mini extruder (Avanti Polar Lipids, USA).

### Preparation of PCA

To prepare the PCA, the mixture of 100 μg AuNs, CDDP (20 μg dispersed in PBS) and PM was sonicated for 0.5 h. Thereafter, a polycarbonate membrane containing 200 nm pore size was used for extruding the mixture 10 times using an extruder. The final mixture was centrifuged and washed with PBS to remove the residual free materials.

### Characterization of the PCA Nanoparticles

The morphology structures of AuNs and PCA nanoparticles were observed by the TEM (JEOL-2100). Hydrodynamic diameter and zeta potential were detected by the dynamic light scattering (Nano-ZS ZEN3600). Drug loading efficiency (DLE) = (weight of loaded drug/weight of feeding drug) × 100%.

### Drug Release Studies

1mg of PCA were dispersed in 1ml PBS solution. The solution was exposed to X-ray irradiation (2 Gy) for 5min. At the given time points, the released CDDP was measured by High Performance Liquid Chromatography (HLPC). No X-ray irradiation was used as a control group.

### Cell Lines and Animal Model

4T1 mouse breast cancer cell lines were provided by the College of Life Science of Wuhan University. All the cells were cultured in the standard cell medium recommended by American Type Culture Collection. Female Balb/c mice aged 4-5 weeks were purchased from Vital River Company (Beijing, China). 5×10^6^ 4T1 cells suspended in 100 μL PBS were subcutaneously injected into each mouse to establish the tumor models. After the tumor volume reached around 200 mm^3^, the tumor bearing mice were used for further experiments. The animal experiments were carried out according to the protocol approved by the Ministry of Health in the People’s Republic of PR China and were approved by the Administrative Committee on Animal Research of the Wuhan University.

### Western Blotting for the Key Proteins in PM and PCA

The total cellular protein in PM and PCA were extracted using a protein extraction kit (Dingguo, China). The extracted proteins were separated using SDS-PAGE electrophoresis. After electrophoresis, the gel was treated with Coomassie blue staining. Extraction of protein for western blot was performed as described above. The proteins were then transferred onto polyvinylidene fluoride (PVDF) membranes (BioRad). This was followed by a blocking step for 1 h with 5% skim milk, and then the membrane was incubated with the primary antibody against P-selectin (Proteintech) overnight at 4°C using Na,K-ATPase as the control. Finally, the membrane was incubated with the secondary antibody for 1 h at room temperature.

### *In Vitro* Tumor-Specific Uptake

At first, Dil labeled RCA and PCA (Containing 100 μg/mL AuNs) were incubated with 4T1 cells for 2 h at 37°C. The cells were then washed with PBS several times, fixed with PFA for 30 min at room temperature, stained with Lyso-Tracker Green and then imaged by using a fluorescence microscope (IX81, Olympus, Japan). The fluorescence intensity was measured by IamgeJ software.

### γ-H_2_AX Immunofluorescence Analysis

4T1 cells were seeded in 24-well plates and then cultured for 24 h at 37°C under hypoxia condition. Then the cells were treated with 1) Control (PBS); 2) Radiotherapy (RT, 2Gy); 3) PCA; 4) PA + RT; 5) PCA + RT. The AuNs concentration was 200 μg/mL in groups 3, 4, and 5. After RT treatment for 2 h, the cells were fixed with 4% paraformaldehyde for 10 min, rinsed with PBS, permeabilized with methanol for 15 min at -20°C and then rinsed with PBS again. Then the cells were exposed to a blocking buffer (1% bovine serum albumin (BSA) in PBS solution) for 1 h at room temperature and further incubated with anti- phospho-histone γ-H2AX mouse monoclonal antibody (dilution 1:500) overnight at 4°C. After washing with PBS, the cells were incubated with Cy5-conjugated sheep anti-mouse secondary antibody (dilution 1:500) for 1 h at room temperature. Excess antibody was removed by rinsing the coverslips in PBS. Cell nuclei were stained by DAPI for 5 min at room temperature. The cells were imaged *via* confocal fluorescence microscopy (IX81). Quantitative analysis of γ-H2AX foci density (foci/100 μm2) was performed by automatic counting using the ImageJ software for n = 100 cells in each treatment group.

### Clonogenic Assay

4T1 cells were seeded in 6-well plates with a different amount (125, 250, 500, 1000 and 2000) per well and incubated at 37°C for 24 h Then the cells were treated with five groups: 1) Control (PBS); 2) Radiotherapy (RT, 2Gy); 3) PCA; 4) PA + RT; 5) PCA + RT. The AuNs concentration was 200 μg/mL in groups 3, 4, and 5. After that, cells were washed with PBS and fresh medium was replaced every three days for 10 days. The colonies were fixed by 4% paraformaldehyde and then stained with Giemsa dye. Only colonies containing at least 50 cells were counted. At last, an evaluation of the effects of different treatments was conducted by counting the survival fraction of the colonies. Each treatment was performed in triplicate.

### *In Vivo* Pharmacokinetics and Distribution Study

4T1 tumor bearing BALB/c mice (n = 3) received an intravenous (i.v.) injection of 100 μL PBS containing RCA, or PCA at the concentration of 1 mg/kg CDDP. At various time points after the injection (i.e.,0.5, 1, 2, 4, 8, 16, 24, and 48 h), 20 μL blood was collected from the tail veins, treated with aqua regia, and then on heated at 70°C until to obtain clear solutions. And the resultant mixture was left standing still at room temperature for 12 h and then kept in oil bath at 70°C for 6 h to remove acids, yielding samples for Pt content quantification by using an ICP-AES (iris Intrepid II XSP, Thermo Elemental, USA). To study the biodistribution of particles in various organs, at 24 h after the injection, all mice were euthanized and their hearts, livers, spleens, kidneys, lungs, and tumors were carefully collected, weighted, and finally quantitatively analyzed with ICP-AES as described above.

### *In Vivo* Antitumor Study

1×10^6^ 4T1 cells suspended in 100 μL PBS were subcutaneously injected into each mouse to establish the tumor models. When tumor size reached approximately 200 mm3, the mice were divided randomly into 5 groups (each group included 5 mice): 1) Control (PBS); 2) Radiotherapy (RT, 2Gy); 3) PCA; 4) PA + RT; 5) PCA + RT. The CDDP dose was 1mg/kg in groups 3 and 5. The treatment was conducted every 5 days for 15 days. Mice body weight and tumor volume in all groups were monitored every 2 days. A caliper was employed to measure the tumor length and tumor width and the tumor volume was calculated according to the following formula. Tumor volume = tumor length × tumor width^2^/2. After 16 days of treatment, all the mice were sacrificed. Their blood samples and major organs (i.e., hearts, livers, spleens, lungs, and kidneys) were collected. Three important hepatic indicators (i.e., ALT: alanine aminotransferase, AST: aspartate aminotransferase, and ALP: alkaline phosphatase) and two indicators for kidney functions (i.e., BUN: blood urea nitrogen and CRE: creatinine) were measured by using a blood biochemical autoanalyzer (7080, HITACHI, Japan). And the tumor tissues were weighed, and fixed in 4% neutral buffered formalin, processed routinely into paraffin, and sectioned at 4 μm. Then the sections were stained with hematoxylin and eosin (H&E) staining and finally examined by using an optical microscope (BX51, Olympus, Japan). Part of their organs were stained with H&E and examined as described above.

### Statistical Analysis

Data analyses were conducted using the GraphPad Prism 5.0 software. Significance between every two groups was calculated by the Student’s t-test. *P < 0.05, **P < 0.01, ***P < 0.005.

## Data Availability Statement

The original contributions presented in the study are included in the article/**Supplementary Material**. Further inquiries can be directed to the corresponding authors.

## Ethics Statement

The animal experiments were carried out according to the protocol approved by the Ministry of Health in the People’s Republic of PR China and were approved by the Administrative Committee on Animal Research of the Wuhan University.

## Author Contributions

MC and ZB: analysis. MC: drafting. DJ and HQ: interpretation of data. PW: revising. All authors contributed to the article and approved the submitted version.

## Funding

We greatly acknowledged the financial support from the National Natural Science Foundation of China (Grant No. 12005158). This work was supported by the Elekta-Wuhan University Medical Physics Teaching and Research Fund (250000200).

## Conflict of Interest

The authors declare that the research was conducted in the absence of any commercial or financial relationships that could be construed as a potential conflict of interest.

## Publisher’s Note

All claims expressed in this article are solely those of the authors and do not necessarily represent those of their affiliated organizations, or those of the publisher, the editors and the reviewers. Any product that may be evaluated in this article, or claim that may be made by its manufacturer, is not guaranteed or endorsed by the publisher.
