# Supplementary material for: Platelet Membranes Coated Gold Nanocages for Tumor Targeted Drug Delivery and Amplificated Low-Dose Radiotherapy
Source: Front Oncol. 2021 Nov 24;11:793006. doi: 10.3389/fonc.2021.793006 (PMC8651991; doi:10.3389/fonc.2021.793006)
Supplement: Supplementary file 1 [file DataSheet_1.docx]

**Experimental section**

**Materials and reagents.**

Deionized (DI) water was obtained by an 18 MΩ cm (SHRO-plus DI) system. Phosphate buffer solution (PBS) and bovine serum albumin (BSA) were purchased from Thermo-Fisher (USA). AuNs were obtained from XFNano (Nanjing, China). , 1’-dioctadecyl-3, 3, 3’, 3’-tetramethylindocarbocyanine perchlorates (Dil) was obtained from Sigma-Aldrich (USA). The γ-H2AX antibody was purchased from Abcam Company. The other reagents used in this work were purchased from Sinopharm Chemical Reagent (China) and Aladdin-Reagent (China).

**Preparation and Characterization of PLT-Vesicles (PM)**

Platelets (PLTs) from whole blood were isolated through gradient centrifugation. 10 mL mice whole blood was centrifuged at 100 × g for 20 min with no brake. Afterward, the supernatant was centrifuged at 800 × g for 20 min. The PLTs were washed by PBS and centrifuged repeatedly. The preparation processes were monitored using a conventional microscope (IX71, Olympus, Japan). PLT membranes were derived by a repeated freeze–thaw process. Aliquots of PLT suspensions were first frozen at −80 °C, thawed

at room temperature, and pelleted by centrifugation at 4000 × g for 3 min. After three repeated washes with PBS mixed with protease inhibitor tablets, the pelleted PLT membranes were suspended in water, sonicated in a capped glass vial for 5 min using a bath sonicator at a frequency of 53 kHz and a power of 100 W, and then extruded sequentially through 400 and 200 nm polycarbonate porous membranes on a mini extruder (Avanti Polar Lipids, USA).

**Preparation of PCA**

To prepare the PCA, the mixture of 100 μg AuNs, CDDP (20 μg dispersed in PBS) and PM was sonicated for 0.5 h. Thereafter, a polycarbonate membrane containing 200 nm pore size was used for extruding the mixture 10 times using an extruder. The final mixture was centrifuged and washed with PBS to remove the residual free materials.

**Characterization of the PCA nanoparticles**

The morphology structures of AuNs and PCA nanoparticles were observed by the TEM (JEOL-2100). Hydrodynamic diameter and zeta potential were detected by the dynamic light scattering (Nano-ZS ZEN3600). Drug loading efficiency (DLE) = (weight of loaded drug/weight of feeding drug) × 100%.

**Drug release studies**

1mg of PCA were dispersed in 1ml PBS solution. The solution was exposed to X-ray irradiation (2 Gy) for 5min. At the given time points, the released CDDP was measured by High Performance Liquid Chromatography (HLPC). No X-ray irradiation was used as a control group.

**Cell lines and animal model.**

4T1 mouse breast cancer cell lines were provided by the College of Life Science of Wuhan University. All the cells were cultured in the standard cell medium recommended by American Type Culture Collection. Female Balb/c mice aged 4-5 weeks were purchased from Vital River Company (Beijing, China). 5×10^6^ 4T1 cells suspended in 100 μL PBS were subcutaneously injected into each mouse to establish the tumor models. After the tumor volume reached around 200 mm^3^, the tumor bearing mice were used for further experiments. The animal experiments were carried out according to the protocol approved by the Ministry of Health in the People’s Republic of PR China and were approved by the Administrative Committee on Animal Research of the Wuhan University.

**Western blotting for the key proteins in PM and PCA**

The total cellular protein in PM and PCA were extracted using a protein extraction kit (Dingguo, China). The extracted proteins were separated using SDS-PAGE electrophoresis. After electrophoresis, the gel was treated with Coomassie blue staining. Extraction of protein for western blot was performed as described above. The proteins were then transferred onto polyvinylidene fluoride (PVDF) membranes (BioRad). This was followed by a blocking step for 1 h with 5% skim milk, and then the membrane was incubated with the primary antibody against P-selectin (Proteintech) overnight at 4 °C using Na,K-ATPase as the control. Finally, the membrane was incubated with the secondary antibody for 1 h at room temperature.

***In vitro* tumor-specific uptake.**

At first, Dil labeled RCA and PCA (Containing 100 μg/mL AuNs) were incubated with 4T1 cells for 2 h at 37 °C. The cells were then washed with PBS several times, fixed with PFA for 30 min at room temperature, stained with Lyso-Tracker Green and then imaged by using a fluorescence microscope (IX81, Olympus, Japan). The fluorescence intensity was measured by IamgeJ software.

**γ-H_2_AX immunofluorescence analysis.**

4T1 cells were seeded in 24-well plates and then cultured for 24 h at 37 °C under hypoxia condition. Then the cells were treated with 1) Control (PBS); 2) Radiotherapy (RT, 2Gy); 3) PCA; 4) PA + RT; 5) PCA + RT. The AuNs concentration was 200 μg/mL in groups 3, 4, and 5. After RT treatment for 2 h, the cells were fixed with 4% paraformaldehyde for 10 min, rinsed with PBS, permeabilized with methanol for 15 min at -20 °C and then rinsed with PBS again. Then the cells were exposed to a blocking buffer (1% bovine serum albumin (BSA) in PBS solution) for 1 h at room temperature and further incubated with anti- phospho-histone γ-H2AX mouse monoclonal antibody (dilution 1:500) overnight at 4 °C. After washing with PBS, the cells were incubated with Cy5-conjugated sheep anti-mouse secondary antibody (dilution 1:500) for 1 h at room temperature. Excess antibody was removed by rinsing the coverslips in PBS. Cell nuclei were stained by DAPI for 5 min at room temperature. The cells were imaged via confocal fluorescence microscopy (IX81). Quantitative analysis of γ-H2AX foci density (foci/100 μm2) was performed by automatic counting using the ImageJ software for n = 100 cells in each treatment group.

**Clonogenic assay.**

4T1 cells were seeded in 6-well plates with a different amount (125, 250, 500, 1000 and 2000) per well and incubated at 37 ℃ for 24 h Then the cells were treated with five groups: 1) Control (PBS); 2) Radiotherapy (RT, 2Gy); 3) PCA; 4) PA + RT; 5) PCA + RT. The AuNs concentration was 200 μg/mL in groups 3, 4, and 5. After that, cells were washed with PBS and fresh medium was replaced every three days for 10 days. The colonies were fixed by 4% paraformaldehyde and then stained with Giemsa dye. Only colonies containing at least 50 cells were counted. At last, an evaluation of the effects of different treatments was conducted by counting the survival fraction of the colonies. Each treatment was performed in triplicate.

***In vivo* pharmacokinetics, and distribution study**

4T1 tumor bearing BALB/c mice (n = 3) received an intravenous (i.v.) injection of 100 μL PBS containing RCA, or PCA at the concentration of 1 mg /kg CDDP. At various time points after the injection (i.e.,0.5, 1, 2, 4, 8, 16, 24, and 48 h), 20 μL blood was collected from the tail veins, treated with aqua regia, and then on heated at 70 °C until to obtain clear solutions. And the resultant mixture was left standing still at room temperature for 12 h and then kept in oil bath at 70 °C for 6 h to remove acids, yielding samples for Pt content quantification by using an ICP-AES (iris Intrepid II XSP, Thermo Elemental, USA). To study the biodistribution of particles in various organs, at 24 h after the injection, all mice were euthanized and their hearts, livers, spleens, kidneys, lungs, and tumors were carefully collected, weighted, and finally quantitatively analyzed with ICP-AES as described above.

***In vivo* antitumor study.**

1×10^6^ 4T1 cells suspended in 100 μL PBS were subcutaneously injected into each mouse to establish the tumor models. When tumor size reached approximately 200 mm3, the mice were divided randomly into 5 groups (each group included 5 mice): 1) Control (PBS); 2) Radiotherapy (RT, 2Gy); 3) PCA; 4) PA + RT; 5) PCA + RT. The CDDP dose was 1mg/kg in groups 3 and 5. The treatment was conducted every 5 days for 15 days. Mice body weight and tumor volume in all groups were monitored every 2 days. A caliper was employed to measure the tumor length and tumor width and the tumor volume was calculated according to the following formula. Tumor volume = tumor length × tumor width^2^ / 2. After 16 days of treatment, all the mice were sacrificed. Their blood samples and major organs (i.e., hearts, livers, spleens, lungs, and kidneys) were collected. Three important hepatic indicators (i.e., ALT: alanine aminotransferase, AST: aspartate aminotransferase, and ALP: alkaline phosphatase) and two indicators for kidney functions (i.e., BUN: blood urea nitrogen and CRE: creatinine) were measured by using a blood biochemical autoanalyzer (7080, HITACHI, Japan). And the tumor tissues were weighed, and fixed in 4% neutral buffered formalin, processed routinely into paraffin, and sectioned at 4 μm. Then the sections were stained with hematoxylin and eosin (H&E) staining and finally examined by using an optical microscope (BX51, Olympus, Japan). Part of their organs were stained with H&E and examined as described above.

**Statistical analysis.**

Data analyses were conducted using the GraphPad Prism 5.0 software. Significance between every two groups was calculated by the Student’s t-test. *P < 0.05, **P < 0.01, ***P < 0.005.


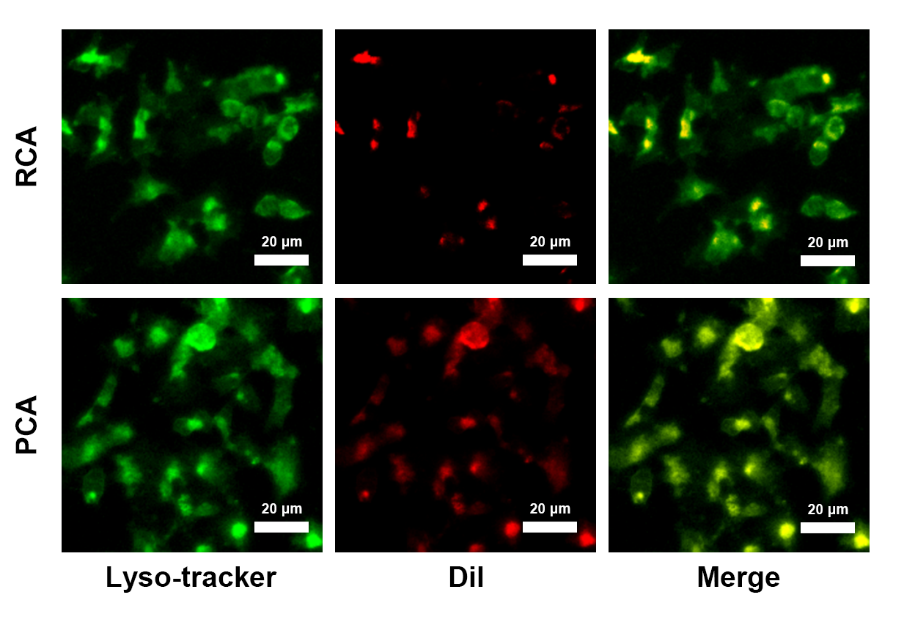


Figure S1. Co-localization of Lyso-Tracker Green FM (green) and Dil (red) for RCA and PCA over time in 4T1 tumor cells.
